# Synthesis and Mechanical Characterization of Binary and Ternary Intermetallic Alloys Based on Fe-Ti-Al by Resonant Ultrasound Vibrational Methods

**DOI:** 10.3390/ma11050746

**Published:** 2018-05-07

**Authors:** Daoud Chanbi, Erick Ogam, Sif Eddine Amara, Z. E. A. Fellah

**Affiliations:** 1 Laboratoire d’Electrochimie, Corrosion, Métallurgie et Chimie Minérale, Université des Sciences et de la technologie de Houari Boumediene, BP 32 El Alia 16111 Bab Ezzouar, Algeria; daoudchanbi@gmail.com (D.C.); samara@usthb.dz (S.E.A.); 2 Laboratoire de Mécanique et d’Acoustique, CNRS, UPR 7051, Aix-Marseille Univ, Centrale Marseille, F-13453 Marseille CEDEX 13, France; fellah@lma.cnrs-mrs.fr

**Keywords:** binary Fe-Ti, Ti-Al, Fe-Al and ternary Fe-Ti-Al alloys, resonant ultrasound vibration, elastodynamic model, first principles calculations, inverse problem

## Abstract

Precise but simple experimental and inverse methods allowing the recovery of mechanical material parameters are necessary for the exploration of materials with novel crystallographic structures and elastic properties, particularly for new materials and those existing only in theory. The alloys studied herein are of new atomic compositions. This paper reports an experimental study involving the synthesis and development of methods for the determination of the elastic properties of binary (Fe-Al, Fe-Ti and Ti-Al) and ternary (Fe-Ti-Al) intermetallic alloys with different concentrations of their individual constituents. The alloys studied were synthesized from high purity metals using an arc furnace with argon flow to ensure their uniformity and homogeneity. Precise but simple methods for the recovery of the elastic constants of the isotropic metals from resonant ultrasound vibration data were developed. These methods allowed the fine analysis of the relationships between the atomic concentration of a given constituent and the Young’s modulus or alloy density.

## 1. Introduction

Iron and aluminium based intermetallic alloys are widely employed today because of their tribo-mechanical properties providing them with exceptional wear, corrosion resistance and greater hardness. They have high elasticity moduli and are highly resistant to oxidation at high temperatures [1,2].

The intermetallic alloys that are intensively studied today are those made from the compounds TiAl, FeAl, and FeTi. The FeTi alloy is of interest to the automotive and aerospace industries and in bio-mechanical applications due to their low density, high strength and ductility. They also have high elastic constants at room temperature [3].

FeAl alloys have excellent resistance to oxidization, sulphating or fuel temperature environments up to 1000 °C and good resistance to corrosion [4]. These mechanical properties together with their relatively low density (<6 g/cm^3^) and elasticity modulus of 205 GPa [5,6], make them potential candidates for the manufacture of structural parts, including in aggressive environments for applications like heat exchangers, engine or turbine components.

TiAl alloys have a fully lamellar microstructure and offer a good balance between all the mechanical properties such as elasticity at room temperature [7,8]. The mechanical properties of ternary Fe-Ti-Al alloys are not only influenced by the amount of Al or Ti contained in the alloy, but also by the microstructure formed in iron-rich or titanium-rich intermetallic alloys [9].

The main objective of this work was to study the variation of the mechanical moduli with the change in the stoichiometric compositions of binary (FeAl, TiAl and FeTi) alloys as well as the influence of addition of a third element such as (Ti, Fe, Al) to form an intermetallic ternary alloy FeTiAl with different chemical compositions. This variation of chemical composition allows the analysis of the modification of Young’s modulus as a function of the crystallographic structure.

The base alloys studied, the phases, prototypes, space groups, the crystal structures and the literature references are tabulated in Table 1. The titanium rich phase ${\mathrm{Ti}}_{3}\mathrm{Al}$ is absent from the aluminium rich phases (TiAl and ${\mathrm{TiAl}}_{2}$) of the TiAl base alloy studied. This is because the field of study was limited to the domain (${\mathrm{Ti}}_{45}{\mathrm{Al}}_{55}$, ${\mathrm{Ti}}_{55}{\mathrm{Al}}_{45}$) in weight percentage (${\mathrm{Ti}}_{32}{\mathrm{Al}}_{68}$, ${\mathrm{Ti}}_{40}{\mathrm{Al}}_{60}$ in atomic percentage) (see phase diagram in reference [10]). The superlattices resulting from the phase transformations (when atoms periodically arrange themselves into a specific ordered array) are reported using the Strukturbericht symbols [11] (L1_0_, L2_1_, B2) and their prototypes (CsCl, ${\mathrm{MgZn}}_{2}$, AuCu, ${\mathrm{HfGa}}_{2}$) are also given. The CsCl (B2) Structure is diatomic with equal numbers of atoms of each type. The L1_0_ structure is a tetragonal distortion of the fcc structure. In the Heusler (L2_1_) Structure, all of the atoms are located on the sites of a body centered cubic lattice. The Mg atoms of the ${\mathrm{MgZn}}_{2}$ hexagonal Laves Structure (C14) are located on the sites of the hexagonal diamond structure.

Several theoretical and experimental studies are available for the determination of the elastic constants of metals and alloys. If the equation of interatomic potential is known, then the elastic constants can be predicted theoretically from the first principles (ab initio) using the density functional theory (DFT) implemented in a software like Quantum Espresso [16] (QE) in combination with Exciting code [17]. The DFT is mostly used for quantum calculations of the electronic structure of solids. This approach was employed in this study to explore the relationship between the chemical composition and elastic properties of the monocrystalline elastic components of the alloys [18].

Young’s modulus can be recovered using measurements of the time of flight (TOF) of ultrasonic waves propagating in the material [19] to retrieve the wave velocity; however, this requires a big test specimen in the form of cylinders, rectangular parallelepiped or rods. This poses a problem when the materials the samples are made of are rare or expensive. The determination of the TOF from which the speed is calculated is not easy either. First, the types of waves (modes propagating in the material) must be identified in the signal before the primary and secondary waves can be chosen. The TOF is usually calculated using the signal peak-to-peak amplitude measurement method or the last zero crossing detection method [20] or through wavelet signal processing methods [21,22].

Resonant ultrasound vibration spectroscopy (RUVS) is an accurate and effective method used to infer material properties, especially elastic properties, from normal modes of isotropic or anisotropic solid materials [23,24,25,26]. The RUVS consists of two parts, the forward or interaction model and the experimental setup to generate data to be used in the inverse problem to infer material properties.

The main advantage of RUVS as a means of retrieving elasticity properties is that the full isotropic elastic tensor of the material can be evaluated from a single sample in a single test configuration [27]. Resonant ultrasonic spectroscopy is now accepted as a useful technique to determine the elastic constants of materials, particularly of small solid crystals [28].

Most of the reported RUVS methods have been extensions of the Demarest’s theory of cube resonance. In this theory, the Rayleigh–Ritz method of eigenvalue approximation is employed to obtain solutions for the free vibration of the elastic solid [29,30,31]. The experimental data used in these methods for the characterization of the elastic body often employ small cubic or spherical specimens. The limitations of using only simple geometries having tractable analytical numerical solutions have been overcome with the adoption of the three-dimensional finite element as an interaction model [32].

The excitation/sensing transducers employed are frequently of the dilatational or shear wave types. Resonance frequencies and ultrasonic attenuation coefficients have also been obtained using electromagnetic acoustic resonance (EMAR) methods [33,34] in which the exciter is composed of a magnet and an electric coil. These transducers generate waves through magnetic field interaction (the alternating current (AC) magnetic field of the coil and the static or quasi-static magnetic field produced by the magnet). The AC current in the electric coil generates eddy current on the surface of the material. The vibration is then induced by the Lorentz force mechanism when the material is conductive (metallic).

In this study, a modified resonant vibration spectroscopy method, in which the specimen is considered isotropic and the transducers and sensor are composed of thin piezoelectric discs, is developed.

The method of synthesis of the alloys is detailed in Section 2.1. The experimental test-rigs employed to generate data from which Young’s moduli of a series of binary and ternary alloys, Fe, Ti and Al, are presented in Section 3. Two different experimental test-rigs are presented, the first one uses a classical RUVS configuration composed of shear wave contact transducers (Section 3.1). The second, developed to overcome the weakness of the first one and improve data precision, employs thin piezoelectric discs [35] (Section 4). In Section 5, the generated data is associated with two elastodynamic models to recover the elastic constants of the different metallic alloy materials. In a last step, the effect of the chemical composition on the elasticity, and the comparison of the mechanical moduli obtained according to the percentages of the chemical compositions present in each sample are studied.

## 2. Experimental Techniques and Configurations

### 2.1. Fabrication of Binary and Ternary Alloy Samples

All the binary and ternary alloys (Ti_*x*_Fe_*y*_, Ti_*x*_Al_*y*_, Fe_*x*_Al_*y*_, Ti_*x*_Al_*y*_Fe_*z*_, where *x*, *y*, *z* are the percentage weight concentrations in the chemical formula) presented in this work were synthesized using an arc furnace (Compact Arc melter MAM-1, Edmund Büler GMBH, Hechingen, Germany), operating under vacuum and flush of argon to avoid oxidation. To ensure uniformity and complete fusion of the alloys, a vacuum was applied, then argon was flushed and this procedure was repeated five times for each alloy. The alloys were prepared from pellets of pure elements, with purity levels for Fe → 99.98%, Ti → 99.99%, Al → 99.98% (the concentrations are in % weight). All the samples were obtained in disc form (with diameter of 2.0 cm) using the same single copper mold. It should be noted that two types of geometries can be obtained from the furnace molds, in disc or baguette form.

### 2.2. Preparation of the Surfaces and Size of the Alloy Disc Samples

Prior to the experimental analysis, the disc samples were first shaped so that the diameter to thickness ratio was approximately 4 (for simplicity of analysis of the experimental data). They were then polished using sheets of sandpaper with different grit sizes (240, 400, 600, 800 and 1200), resulting in well polished surfaces and also well adjusted sample sizes with diameters of 14 ± 0.5 mm and thicknesses of 3 ± 0.5 mm.

## 3. Experimental Methods for Vibration Spectrum Data Acquisition

Two different experimental setups for acquiring the ultrasonic vibrational spectra of the disc samples were developed.

### 3.1. Configuration Using Shear Wave Transducers

**Figure 1:**
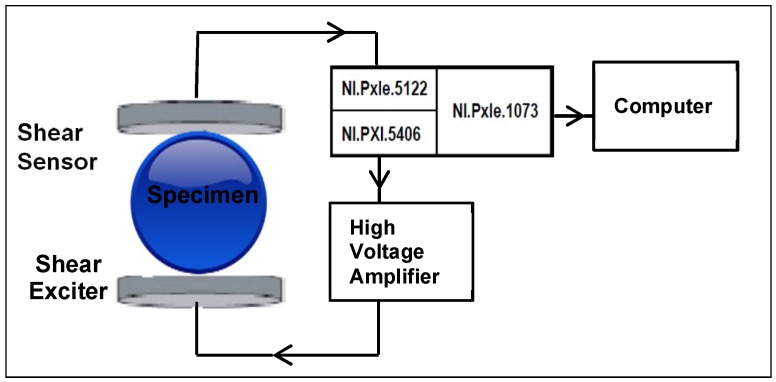
The first vibration spectroscopy experiment using shear wave transducers. The diagram depicts the setup employed.

This is the classical way of acquiring resonant ultrasonic vibration spectroscopy data. In our setup, this system consisted of two normal incidence shear wave transducers (Panametrics Videoscan V150, 0.25 MHz, Waltham, MA, USA). The first transducer was applied as exciter and the second one as sensor. The exciter was connected to a high speed high voltage amplifier (WMA-300, Falco Systems, Katwijk, The Netherlands). In order to capture the response signals, the sensor was connected to an NI PXIe-5122 high-resolution oscilloscope slotted into an NI PXI-1073 chassis (National Instruments, Austin, TX, USA). The exciter signal was generated using a 40 MHz Bandwidth, 16-Bit PXI Waveform Generator. The chassis was connected to a computer running the signal processing software called Resonance Inspection Techniques and Analysis (Rita, version 3.1.65), employed to acquire the vibration spectrum. The setup diagram is illustrated in Figure 1.

The alloy disc sample was then placed between the pair of shear wave transducers. The tightening of the sample was done by adjusting the bottom transducer up and down, the upper one remaining fixed (see Figure 1). This was to prevent pressure being applied on the sample by the upper transducer (thus ensuring stress-free boundary conditions). The samples were then excited using continuous harmonic mode and the frequency varied by performing a frequency sweep (30 kHz to 400 kHz in 100 Hz steps). The responses of the samples were captured by the second transducer. The samples were rotated in several directions and at each time their vibration spectrum acquired to check their isotropy.

## 4. Configuration with Piezoelectric Discs

The presence of spurious peaks, high damping of the response (the mechanical attachment of the transducers) and weak presence of the first mode in the spectral response (at around 60 kHz while the central frequency of the transducer is at 0.25 MHz) encouraged us to find a better alternative setup.

**Figure 2:**
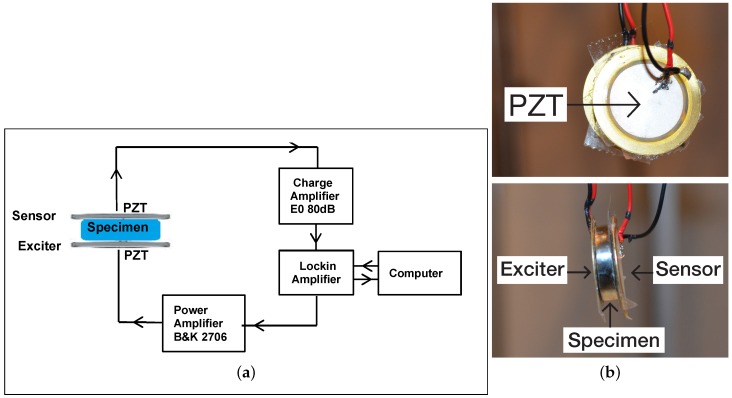
The second vibration spectroscopy experimental setup. (**a**) diagram of the setup employed; (**b**) upper panel, side view photograph of the piezoelectric exciter and sensor, lower panel, front view of the thick disc alloy specimen placed between the piezoelectric exciter and the sensor discs.

The second configuration employed two thin piezoelectric transducer (PZT) discs. The PZT discs were retrieved from piezoelectric audio transducers used as sounders by removing them from the resonators. Since their response characteristics were not know beforehand, three different discs were tested on the specimens and their responses evaluated to get the best choice for the experiment. They were referenced KPEG827, KPEG110, KPEG165 (Kingstate, Taipei, Taiwan) with diameters 25 mm, 20 mm, 10 mm, respectively. Two thin PZT discs were necessary and had the same diameter. The first PZT disc was used as the exciter while the second as sensor to capture the spectral vibration response of the metal sample. The carefully polished metal disc alloy sample was placed between the pair of PZT transducer discs. The PZT disc, Kingstate KPEG 110, 20 mm diameter, gave the best response for the samples tested. The experimental set-up diagram and photograph are shown in Figure 2.

The exciter was driven in discrete frequency steps using the lock amplifier (Signal Recovery 7265 DSP, Oak Ridge, TN, USA). Contact between the two flat surfaces of the sample and the PZT was ensured using thin double sided scotch tape. The sample and the PZT discs taped to it were then suspended to ensure stress-free vibrations without constraint as shown in Figure 2. Scotch tape was cut exactly to fit the PZT disc size in order to limit the impact of damping on the measurements that the scotch tape may add. The sample was excited using continuous harmonic stimulation, realizing a frequency sweep (of frequency, 50–250 kHz in 200 Hz steps). The response was captured by the second piezoelectric disc connected to a low noise amplifier (EO80dB, Ciprian, Grenoble, France). Rotation of the alloy disc sample in several directions was done to verify that the vibration response spectra were the same and thus verify the isotropy of the samples and also to confirm the repeatability and consistency of the experimental data obtained.

## 5. The Ingredients for Estimating Alloy Disc Sample Elastic Moduli

The alloy samples in this study were considered as equivalent single crystal elastic bodies whose mechanical behavior could be captured precisely using the elastodynamic model of an isotropic elastic solid. Two elastodynamic models of vibration were candidates for solving the direct problem. The first model incorporated more physical and geometric information on the sample, but required more intensive computations. The second model was not computationally-intensive and was chosen as candidate to solve the inverse problem, which was the recovery of Young’s moduli and Poisson ratio from the resonance frequencies of the samples. The second model was validated using the first model. The first model was then used to finely adjust those parameters that had been identified by solving the inverse problem.

### 5.1. First Model for Solving the Direct Problem

The choice of the correct model for solving the direct problem is a key issue for the validation of data retrieved from experimental measurements. The inversion process can be one in which a set of computed observable, in this case eigenfrequencies, are used to calculate a succession of trial values of the sought for parameters of the configuration from experimental data. In such a process, there is need to compute the resonance frequencies of a given structure configuration and then compare them to the measured resonance frequencies of the test sample to recover its elastic modulus and Poisson ratio. In this study, the computed eigenfrequencies were obtained using an interaction model based on the finite element method. They were then compared to the experimental resonance frequencies recovered from the measured spectral response. This permitted the retrieval of the optimal values of the elastic moduli and Poisson ratio that were recovered by the second model that follows. The computations were done in three dimensions (3D) using an elastodynamic finite element method (FEM) [36,37] implemented in the commercial software Abaqus (version 6.14, Dassault Systèmes Simulia Corporation, Providence, RI, USA) [38]. This constituted the first interaction model.

### 5.2. Second Model for Solving the Direct Problem

The frequency response for a time harmonic excitation applied to the sample was computed using a quasi analytic model for computing the resonance frequencies of a thick circular disc, a model developed by Martinceck [39] and also adopted in the standard test method [40]. The model equation defines the relationship between the resonance frequency and the elastic properties of the material and the dimensions of the disc shaped sample. It is written as follows:

(1) $$f_{i}=\frac{K_{i}}{2\pi r^{2}}\sqrt{\frac{A}{\rho t}},$$

where $f_{i}$ is the resonance frequency (i=1,2), $K_{i}$ is the geometry factor for the resonance frequency, *r* is the radius of the disc, the disc constant $A=Et^{3}/\left[12\left(1-\nu^{2}\right)\right]$, *E* is Young’s modulus, *t* is the disc thickness, ν is the Poisson ratio and ρ is the density. In this study, the ratio t/r was equal to 0.42.

### 5.3. Solving the Inverse Problem of Vibrational Spectroscopy

The second interaction model equation was rewritten to find Young’s modulus as a function of the resonance frequency. The expression calculated from Equation (1) is rewritten as:

(2) $$E_{i}=\left(48{\left[f_{i}\right]}^{2}\pi^{2}r^{4}\left(1-\nu^{2}\right)\rho \right)/\left({\left[K_{i}\right]}^{2}t^{2}\right).$$

It is a quasi-analytical equation, thus the inversion process is instantaneous. The geometric factors computed for the ratio t/r=0.42 are $K_{1}=4.6356$ and $K_{2}=7.3284$. Poisson ratio read from nomograms [39] in terms of the ratio t/r≈0.4 and the resonance frequency ratio $f_{2}/f_{2}\approx 1.57$ (for most alloys in this study) was ν≈0.27. Poisson ratio was initialized using this value. Once Young’s modulus and Poisson ratio recovered, they were injected into the sophisticated 3D FEM to calculate the resonance frequencies which were in turn compared with the measured frequencies. The values of Young’s modulus and Poisson ratio were further adjusted so that the RUV experimental frequencies and those generated by the 3D FEM matched. These were then the values considered as final.

### 5.4. How to Identify and Classify the Modes of Vibration in the Response Spectrum of the Specimen

The vibration spectroscopy spectrum is composed of a multitude of resonance frequencies of the three-dimensional elastic and isotropic solid body, indicated by peaks. The vibration spectrum peaks correspond to different families of modes (torsional, bending, longitudinal, etc.), which are not easy to locate or pinpoint on the spectral vibrational response [37].

To resolve the issue of identifying the modes of vibration corresponding to the observed spectral vibration peaks, a simple argument is employed. Unless heat treatment and/or and severe plastic deformation techniques are used to improve the Young’s modulus of an alloy, its elastic properties will depend on the crystallographic structure, which depends on the chemical composition of the alloy (see Table 1). The eigenvalues of a disc sample made of a single crystal of one of the components of the metal alloys were computed. This provided an idea of where the first and second modes would be situated in the vibration spectrum. The elastic moduli of the single crystal components from ab initio computation for pure metals that make up the alloy, and also parameters such as density and Poisson ratio were employed. The values for the single crystal were from the literature; therefore, the computations were done to confirm them and evaluate the different first principal DFT method calculation tools. For titanium, the density functional theory using the projector augmented-wave (PAW) method in Quantum Espresso associated with the Exciting [17,41] package was employed. The mechanical parameters were then injected into the 3D finite element model to calculate the eigen-frequencies and plot the mode shapes/deformation.

The identifications of the types of modes was also confirmed theoretically. A 3D elastodynamic finite element direct-solution steady-state dynamic analysis was used to calculate the steady-state dynamic linearized response of the disc submitted to a concentrated harmonic force excitation [42]. The concentrated force was applied at the center of the disc to excite only longitudinal modes. In a second step, the force was applied to the edge of the disc to excite all the modes. This enabled the identification of the types of modes (whether shear or longitudinal) captured in the vibration spectrum.

## 6. Results

### 6.1. Identification and Classification of Vibration Modes in the Spectrum and Validation of the Interaction Model Used for Inversion

**Figure 3:**
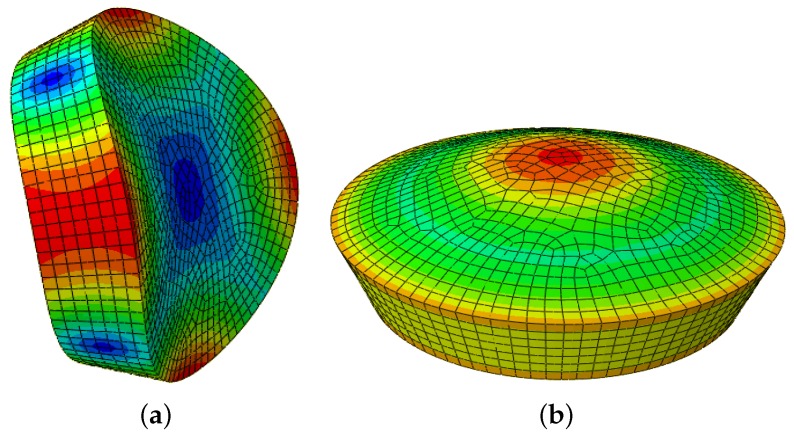
The natural vibration modal deformations of the thick discs, (**a**) the first flexural mode; (**b**) the first compressional mode.

The 3D FEM elastic model of the disc specimen, implemented using Abaqus software, was discretized using 6041, 20-node quadratic hexahedral elements of type C3D20R [36,38,43]. The first flexural and longitudinal mode shapes are depicted in Figure 3. The mode shape deformations induce mechanical deformations in the polarized PZT sensor crystal bonded to the deforming vibrating surface of the disc. The resulting tension and twisting of the sensor leads to the generation of an electric charge. Likewise, when the PZT crystal is submitted to a modulated electrical solicitation, it vibrates and induces a stress wave. The mode shapes showed that the vibrational modes of the disc could be easily detected using the thin PZT disc sensor.

The results of the computation of the resonance frequencies of the single crystal elements are given in Table 2. These results will guide the choice of the first and second resonance family of modes from the spectral vibrational response of the alloy disc specimens.

### 6.2. Validation of the Interaction Model for the Inverse Problem Using Synthetic Data

In order to test the inversion method, the synthetic data of resonance frequencies calculated using the 3D FEM isotropic elastic model were employed. The theoretical Young’s moduli data for the Ti-Al system from reference [44] used in the 3D FEM were computed using the embedded atom method (EAM) type interatomic potentials fitted to ab initio data. The data was generated by density functional calculations, employing the linearized augmented plane wave (LAPW) method within the generalized gradient approximation (GGA). We confirmed the values for titanium using Quantum Espresso associated with the Exciting package. The computation for the mono crystal aluminium required a dense **k**-point mesh of 36×36×36 [41] to achieve reasonable precision therefore necessitating long hours of computation.

Young’s moduli were then recovered using Eqn. 2 of the second interaction model. In this case, the density and Poisson ratio were assumed to be known. The recovered values ($E_{1}^{r}$ and $E_{2}^{r}$ in Table 2) are in good agreement with the ones used for the direct problem (in the 3D FEM interaction model) to generate the resonance frequencies.

### 6.3. Comparison between the Two Experimental Resonance Ultrasound Vibrational Methods

**Figure 4:**
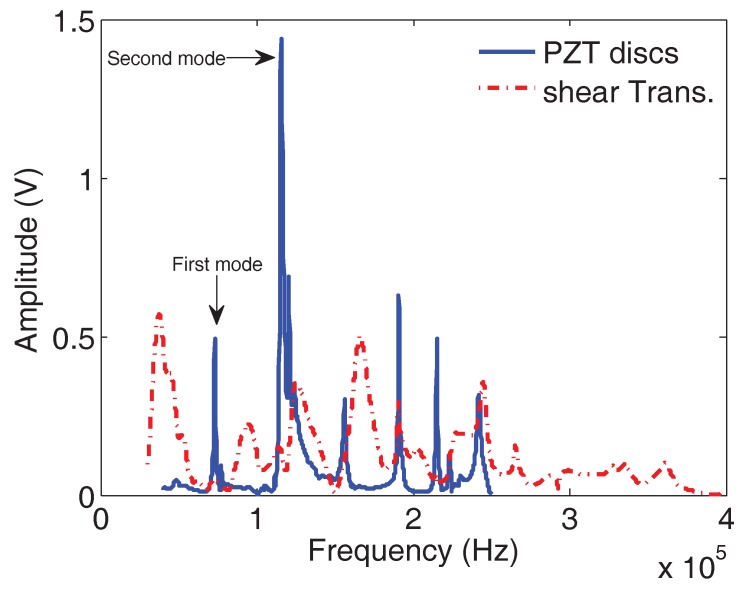
Comparison between the spectra obtained using the shear wave and the piezoelectric disc transducers for the same disc ternary sample ${\mathrm{Fe}}_{63}{\mathrm{Ti}}_{29}{\mathrm{Al}}_{8}$ (composition given in percentage by weight).

**Figure 5:**
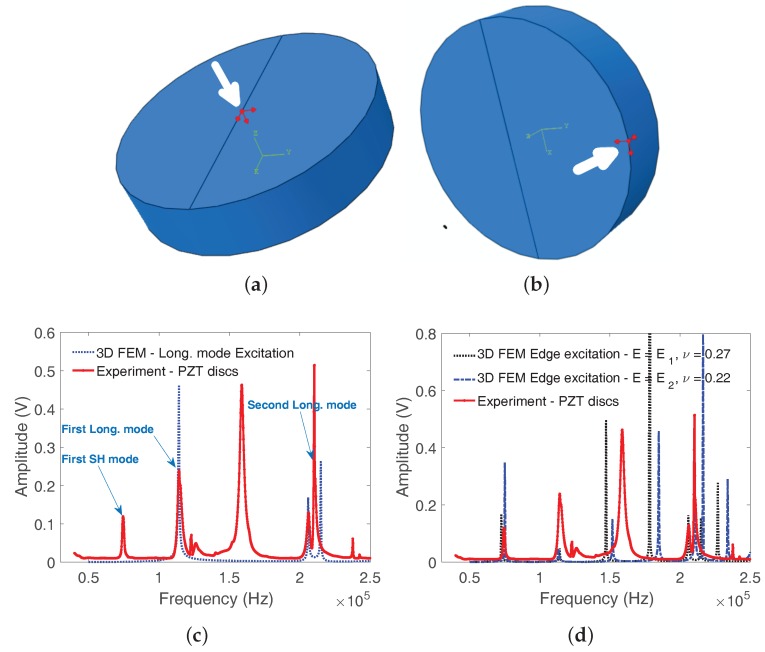
The vibration spectrum obtained using the piezoelectric disc transducers for the ternary disc sample ${\mathrm{Fe}}_{52}{\mathrm{Ti}}_{22}{\mathrm{Al}}_{26}$ (composition given in percentage by weight). (**a**) point concentrated force excitation setup for the longitudinal modes only; (**b**) edge excitation for all modes; (**c**) dotted lines are the 3D finite element method computation of the disc response picked at a node on the opposite face where excitation is applied. The first shear (SH) and Longitudinal (Long.) modes are also shown; (**d**) edge excitation responses showing all the modes with two different values of the pair (*E*, ν).

The vibration spectrum of a ternary alloy disc sample ${\mathrm{Fe}}_{63}{\mathrm{Ti}}_{29}{\mathrm{Al}}_{8}$ (composition given in percentage by weight) obtained using the setup employing shear wave transducers and piezoelectric discs are plotted in Figure 4. The resonance peaks appear better marked and more easily identified in the spectrum obtained using the thin PZT discs than those obtained using the shear wave transducers that have multiple extra peaks (as stated in Section 4). The choice was made to exploit the results obtained using the piezoelectric disc. In order to excite only the longitudinal modes in the 3D FEM direct-solution steady-state dynamic analysis, a point concentrated force $\vec{f}\left(\omega \right)$ was applied at the center of the disc (force whose components in Newton were (0, 0, −1)) (Figure 5a). All the modes were excited by applying a point force $\vec{f}\left(\omega \right)$ (1,−1,−1) at the edge of the disc (Figure 5b). The results of the 3D FEM computation of the disc responses picked at a node on the opposite face of where the forces were applied are also shown for a ternary disc sample ${\mathrm{Fe}}_{52}{\mathrm{Ti}}_{22}{\mathrm{Al}}_{26}$ (composition given in percentage by weight) in Figure 5c,d. The first two longitudinal modes are well identified in the setup with the force applied in the middle of the disc (Figure 5c). The first shear (SH) mode and the other modes are also well identified from the computed response with the force applied at the edge of the disc (Figure 5d). Two responses using the latter configuration were calculated for two sets of Young’s modulus and Poisson ratio to show the variation of the resonance peaks. The good agreement between the computed and measured spectral resonance frequencies confirm that the isotropic elastic model captures well the experimental data.

Young’s moduli retrieved from the vibration spectra resonance frequencies obtained using the thin piezoelectric transducer discs (Table 3) are in good agreement with those obtained using the shear wave transducers (Table 4). They also agree with the values obtained by adjusting the parameters in the 3D finite element model. The recovered Young’s moduli were also compared with those of the same composition found in the literature. There was a good agreement with those calculated using ab initio theory. For example (Table 4), the Young’s modulus of ${\mathrm{Fe}}_{60}{\mathrm{Ti}}_{40}$ (wt. %) was compared with that from reference [50] (difference with E was +0.6 percent). The ab initio computations were done using the DFT method based on the full-potential (linearized) augmented plane-wave ((L)APW) and local orbitals (lo) method implemented in the WIEN2K software (K. Schwartz Technical Universität, Vienna, Austria) [51]. Calculations for ${\mathrm{Ti}}_{45}{\mathrm{Al}}_{55}$ [52] (difference with E was −1.1 percent) were performed using the density functional theory (DFT) based on the projector augmented wave and the generalized gradient approximations (GGA) as implemented in the Vienna Ab initio Simulation Package (VASP) (Universität Wien, Vienna, Austria) [53].

A bigger difference between $E_{2}$ and *E* meant an important adjustment of the latter in the 3D FEM moduli or Poisson ratio. It was found that the chosen initial value of Poisson ratio of ν = 0.27 was slightly underestimated or overestimated.

## 7. Discussion

The recovered Young’s moduli obtained using the spectrum resonance frequencies obtained from the setup using PZT discs ($E_{1}$ and $E_{2}$ matching the frequencies $F_{1}$ and $F_{2}$) were compared with existing published results for the ${\mathrm{Fe}}_{80}{\mathrm{Al}}_{20}$ binary alloy [6] ($E_{1}$ and $E_{2}$ were 201.44 GPa and 199.56, respectively) as well as those for the ${\mathrm{Fe}}_{60}{\mathrm{Al}}_{40}$ alloy (196.37 GPa and 197.73 GPa). The difference with the measured published results (200 GPa [6,55]) does not exceed 1% (Table 3).

The agreement between the values of $E_{1}$ and $E_{2}$ retrieved from the frequencies $F_{1}$ and $F_{2}$ shows that the values of the Poisson ratios are correct, and also confirms the homogeneity of the alloys. Binary alloys ${\mathrm{Ti}}_{55}{\mathrm{Al}}_{45}$ and ${\mathrm{Ti}}_{45}{\mathrm{Al}}_{55}$ were found to have a Young’s modulus of 165.92 GPa and 163.70 GPa, respectively. These values indicate that the alloy that contains more titanium has a greater Young’s modulus. The percentage of titanium in an alloy is proportional to the Young’s modulus of this alloy. These results are in full agreement with the theoretically calculated values found in [52], and with the measured values reported in the literature (between 145 and 175 GPa) in [54,56].

### 7.1. Comparison with Some Published Results in the Literature

It is possible to compare the results obtained by other compositions of the same nature. The results pertaining to alloys ${\mathrm{Fe}}_{60}{\mathrm{Ti}}_{40}$ and ${\mathrm{Fe}}_{50}{\mathrm{Ti}}_{50}$ were compared. Their Young’s moduli were 191.57 GPa and 183.47 GPa, respectively. These values indicate that the effect of adding titanium decreases the Young’s modulus of the Fe-Ti alloy. The values obtained in this study are close to the measured values reported in reference [47] of 190 GPa. However, ternary Fe-Ti-Al alloys with an increasing percentage of iron (47, 50, 52, 57, 63, 68 and 78, weight percentage) have Young’s moduli increasing from 184.04 GPa to 209.56 GPa. They correspond to measured values reported in [57] (varying from 210 to 215 GPa). These values are close to previously obtained Young’s moduli of binary alloys containing iron (FeTi and FeAl). This can be interpreted for an alloy as, the value of its Young’s modulus will approach that of the element in its composition for which the Young’s modulus is the highest.

Properties of intermetallic alloys are almost the same as those of the majority elements in the composition. These properties are enhanced when elements such as aluminum and titanium are added.

### 7.2. The Influence of the Formed Phases and Their Crystallographic Structures on the Mechanical Properties

**Figure 6:**
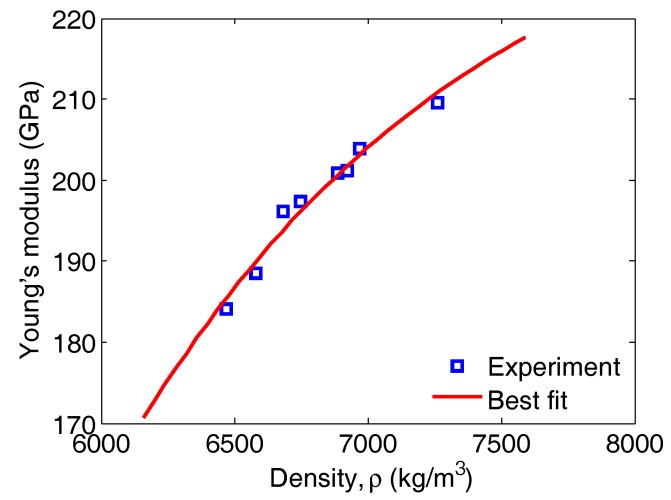
The variation of Young’s modulus, retrieved from data generated by the setup employing the thin piezoelectric disc transducers, versus the ternary Fe-Ti-Al alloy density.

The alloys’ Young’s moduli were plotted against their density. The variation of Young’s modulus for the ternary alloy, Fe-Ti-Al, is depicted in Figure 6. These data were fitted onto an exponential equation of the type E(ρ)=a−bexp(−cρ) (the fitting parameters were a=242.1, b=7263, c=7.5e−4, with 95% confidence bounds). The curve indicates that the increase in Young’s modulus with the increase in density follows a very clear trend and shows the good correlation between density and mechanical properties of metals. The increase in the percentage of the iron quantity in the alloy results in an increase in its density. This explains why an alloy whose composition is 65–99% (atomic percentage) iron maintains satisfactory mechanical properties.

Considering the crystallographic structures in Table 1 of each phase formed in the alloys, we found that the crystallographic structure varied with the percentages of the chemical compositions. This explains the variation of Young’s modulus with crystallographic structure, for example, Young’s modulus for the phase ${\mathrm{Fe}}_{2}\mathrm{Ti}$ that matches the C14 structure is greater than that of the phase FeTi, which corresponds to the B2 structure. As demonstrated previously by the curve in Figure 6 showing the evolution of Young’s modulus against the percentage of iron, the C14 structure contains more iron. This gives a clear interpretation that Young’s modulus changed with the change of the crystallographic structure.

**Figure 7:**
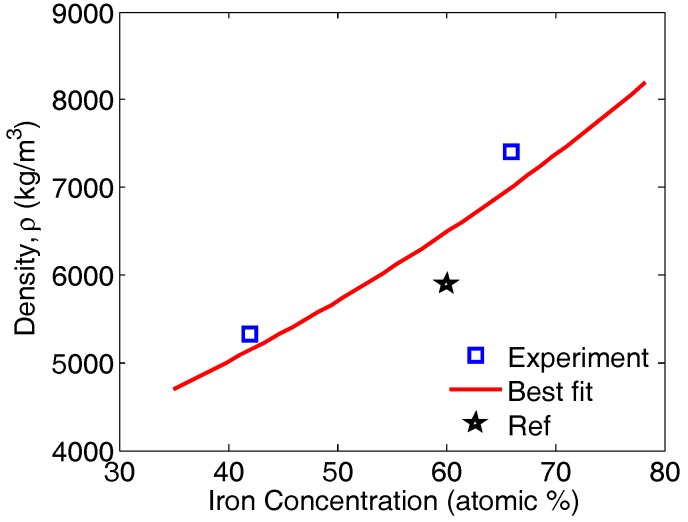
The variation of the density of the binary FeAl alloy as a function of the iron content. The data point shown by the pentagram is from reference [6].

The curve (Figure 7) for binary FeAl alloy with points representing the mass density as a function of the atomic percentage was also plotted for ${\mathrm{Fe}}_{66}{\mathrm{Al}}_{34}$ (atomic percentage) and ${\mathrm{Fe}}_{42}{\mathrm{Al}}_{58}$ (atomic percentage) (see Table 3). In order to fit the data for the two binary alloys with an exponential equation of the type *E*(atomic%) = *a* exp (*b* atomic%), the two points were not enough, therefore data from reference [6] for ${\mathrm{Fe}}_{60}{\mathrm{Al}}_{40}$ (atomic percentage) was borrowed (i.e, ρ= 5900 kg/m^3^). The parameters that fitted the exponential equation were as follows; a=2999, b=2.1e2 (with 95% confidence bounds). Therefore, with the knowledge of iron concentration in the alloy, the density of the FeAl alloy can be determined from the curve or predicted from the equation. This works perfectly for the FeAl alloys in Table 3 and Table 4.

**Figure 8:**
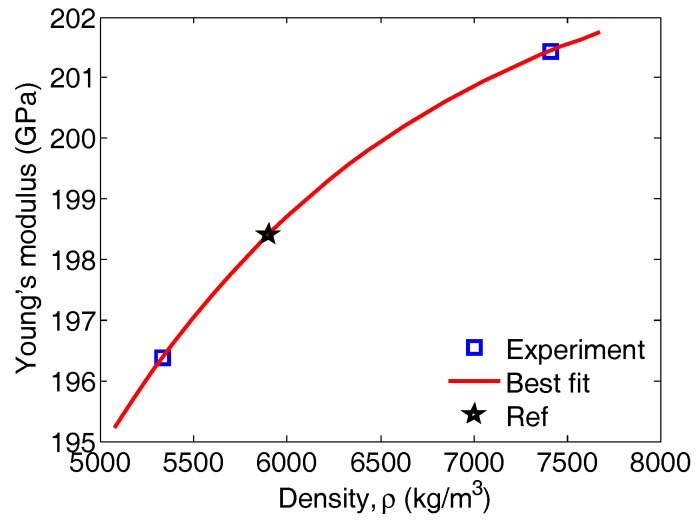
The variation of the Young’s modulus of the binary alloy FeAl as a function of its density. The data were generated by the setup employing the thin PZT disc transducers. The data point shown by the pentagram is from reference [6].

Similarly, a curve using data for the two previous alloys’ Young’s moduli and densities was plotted (Figure 7). As remarked a priori, only two data points were available, and so the third one was borrowed from the same reference [6] (E=200 GPa, ρ= 5900 kg/m^3^) in order to fit the data to an exponential equation of the type E(ρ)=a−bexp(−cρ) (constants obtained a=203.6, b=161.8, c=5.8e−4, with 95% confidence bounds). In summary, if the Fe concentration in the FeAl alloy is known, then the density of the alloy can be retrieved from the projection on the curve in Figure 7 or its corresponding equation. Then, its Young’s modulus can be recovered from the projection of the density on the curve in Figure 8 or its exponential equation.

## 8. Conclusions

Experimental methods using vibrational resonance ultrasound were developed to generate real data pertaining to the resonant frequencies of intermetallic alloy samples, each in the form of a thick disc. The RUV data was combined with two theoretical interaction elastodynamic models (Quasi analytic eigen-frequency model for disc and 3D FEM) to recover the mechanical moduli of intermetallic binary (Fe-Al, Fe-Ti and Ti-Al) and ternary (Fe-Ti-Al) alloys having different chemical concentrations. Elastic constants of intermetallic alloys obtained using theoretical models employing the first principle (ab initio) methods based on DFT calculations were compared with those recovered from the experimental data obtained in this study. There was an excellent agreement between the present results and those reported. The measurement results also highlighted the isotropy of the alloy materials. This was indicated by the distance between the values of the first and second resonance mode frequencies obtained by the two RUV experimental methods.

On the other hand, an overall increase in Young’s modulus with the density values of the samples was observed. A clear dependence of Young’s modulus and density on the chemical composition was also noted.

An empirical exponential equation relating the ternary Fe-Ti-Al alloy density to its Young’s modulus was found. The first equation for the binary Fe-Al alloy provided the relationship for predicting the density of the alloy when the concentration of iron is known. This was followed by a second equation relating the Young’s modulus of the alloy to its density. These findings provide new empirical predictive relationship equations for these alloys.

The PZT disc RUV method was the preferred method for recovering Young’s moduli of binary and ternary alloys at ambient temperature because the resonance peaks were clear, with all resonance modes in the frequency spectrum identified using 3D FEM. The lightweight nature (less invasive and low stress application on the specimen) and low cost of the PZT discs give them an advantage over the shear wave transducers.

In order to further our study on the variation of the Young’s modulus based on the crystallographic structures of the alloys and the influence of adding elements such as (Fe, Ti, Al) on the mechanical properties, the scanning electron microscope (SEM) coupled to Energy Dispersive X-ray Spectroscopy (EDS) will be used to determine all the crystallographic structures formed in our alloy phases. This will constitute the second part of this work.

## Figures and Tables

**Table 1:** The observed phase in the binary and ternary systems studied.

Base Alloy Studied	Phases	Prototype	Space Group	Crystal Structure [12]	References
FeTi	FeTi	CsCl	Pm-3m	B2	[10,13,14]
	Fe_2_Ti	MgZn_2_	P6_3_6/mmc	C14	
TiAl	TiAl	AuCu	P4/mmm	L1_0_	[13,14,15]
	TiAl_2_	HfGa_2_	I4_1_/amd	-	
FeAl	FeAl	CsCl	Pm-3m	B2	[10,13,14]
	FeAl_2_	FeAl_2_	P1	-	
FeTiAl	Fe_2_AlTi	-	Pm-3m	L2_1_	[10,13,14]

**Table 2:** Mechanical properties calculated by ab initio (density functional theory), and resonance frequencies of the single crystal metals computed using 3D finite element method ($F_{1}$, $F_{2}$). $E_{1}^{r}$ and $E_{2}^{r}$ are the recovered Young’s moduli (in GPa) using the synthetic resonance frequencies ($F_{1}$, $F_{2}$) and the second interaction model (Equation (2)).

Element	Density (Kg/m^3^)	Young’s Modulus	Poisson Ratio (*ν*)	*F*_1_ (Hz)	*F*_2_ (Hz)	$E_{1}^{r}$	$E_{2}^{r}$
**Fe**	7874 Ref. [45]	212 Ref. [46]	0.27	70480	110577	213.27	210.05
**Ti**	4500 Ref. [47]	114.6 Ref. [44,48]	0.3	67932	109053	111.14	114.60
**Al**	2707 Ref. [45]	69.3 Ref. [45,49]	0.3	68230	109530	67.27	69.36

**Table 3:** The resonance frequencies ($f_{1}$, $f_{2}$) recovered from the vibration spectra obtained using piezoelectric disc transducers and the corresponding Young’s moduli ($E_{1}$, $E_{2}$) retrieved using Equation (2). The final 3D FEM Young’s moduli (*E*) after adjustment to fit experimental resonance frequencies. The difference between $E_{2}$ and *E* is given in percentage. The † indicates ab initio calculated results found in the literature. The values of $K_{1}$ = 4.6356, $K_{2}$ = 7.3284, and the initial ν = 0.27.

Composition	Composition	$f_{1}$	$f_{2}$	Density	$E_{1}$	$E_{2}$	E (Gpa)	$F_{1}$ (Hz)	$F_{2}$ (Hz)	Difference Percentage	Reference
(wt. %)	(at. %)	(Hz)	(Hz)	(Kg/m^3^)	(GPa)	(GPa)	3D FEM	3D FEM	3D FEM	$E_{1}$ − E (%)	E (GPa), ν
${\mathrm{Fe}}_{47}{\mathrm{Ti}}_{23}{\mathrm{Al}}_{30}$	${\mathrm{Fe}}_{35}{\mathrm{Ti}}_{20}{\mathrm{Al}}_{45}$	72,600	114,200	6469	185.92	184.04	184.04	72,682	114000	1.02	
${\mathrm{Fe}}_{50}{\mathrm{Ti}}_{23}{\mathrm{Al}}_{27}$	${\mathrm{Fe}}_{38}{\mathrm{Ti}}_{20}{\mathrm{Al}}_{42}$	73,000	114,600	6580	191.20	188.54	188.54	72,942	114,440	1.41	
${\mathrm{Fe}}_{52}{\mathrm{Ti}}_{22}{\mathrm{Al}}_{26}$	${\mathrm{Fe}}_{40}{\mathrm{Ti}}_{20}{\mathrm{Al}}_{40}$	73,800	116,000	6682	198.44	196.16	196.16	73,883	115,836	1.16	
${\mathrm{Fe}}_{57}{\mathrm{Ti}}_{22}{\mathrm{Al}}_{21}$	${\mathrm{Fe}}_{45}{\mathrm{Ti}}_{20}{\mathrm{Al}}_{35}$	73,800	115,800	6748	199.31	197.42	197.42	73,665	115,637	0.96	
${\mathrm{Fe}}_{63}{\mathrm{Ti}}_{29}{\mathrm{Al}}_{8}$	${\mathrm{Fe}}_{55}{\mathrm{Ti}}_{30}{\mathrm{Al}}_{15}$	73,000	115,600	6888	200.14	200.82	200.82	73,665	115,575	0.34	
${\mathrm{Fe}}_{65}{\mathrm{Ti}}_{20}{\mathrm{Al}}_{15}$	${\mathrm{Fe}}_{56}{\mathrm{Ti}}_{20}{\mathrm{Al}}_{24}$	73,000	115,400	6925	201.22	201.20	201.20	73,450	115,238	0.01	
${\mathrm{Fe}}_{68}{\mathrm{Ti}}_{24}{\mathrm{Al}}_{8}$	${\mathrm{Fe}}_{60}{\mathrm{Ti}}_{25}{\mathrm{Al}}_{15}$	73,000	115,800	6972	202.59	203.97	203.97	73,704	115,638	0.68	
${\mathrm{Fe}}_{78}{\mathrm{Ti}}_{14}{\mathrm{Al}}_{8}$	${\mathrm{Fe}}_{70}{\mathrm{Ti}}_{15}{\mathrm{Al}}_{15}$	72,800	115,200	7263	209.89	209.56	209.56	73,196	114,038	0.16	
${\mathrm{Fe}}_{80}{\mathrm{Al}}_{20}$	${\mathrm{Fe}}_{66}{\mathrm{Al}}_{34}$	70,600	111,200	7412	201.44	199.56	199.56	70,706	110,933	0.94	200 Ref. [6]
${\mathrm{Fe}}_{60}{\mathrm{Al}}_{40}$	${\mathrm{Fe}}_{42}{\mathrm{Al}}_{58}$	82,200	130,400	5330	196.37	197.73	197.73	82,997	130,216	0.69	“
${\mathrm{Fe}}_{60}{\mathrm{Ti}}_{40}$	${\mathrm{Fe}}_{56}{\mathrm{Ti}}_{44}$	70,800	111,600	7050	192.69	191.57	191.57	71,033	111,445	0.58	191.66, ν = 0.287 Ref. [47] †
${\mathrm{Fe}}_{50}{\mathrm{Ti}}_{50}$	${\mathrm{Fe}}_{46}{\mathrm{Ti}}_{54}$	70,000	110,000	6950	185.69	183.47	183.47	70,013	109,845	1.21	182.38, ν = 0.28 Ref. [50] †
${\mathrm{Ti}}_{55}{\mathrm{Al}}_{45}$	${\mathrm{Ti}}_{40}{\mathrm{Al}}_{60}$	88,200	140,000	3880	164.58	165.92	165.92	89,109	139,805	0.81	160–176 Ref. [54]
${\mathrm{Ti}}_{45}{\mathrm{Al}}_{55}$	${\mathrm{Ti}}_{32}{\mathrm{Al}}_{68}$	91,200	144,200	3609	163.37	163.70	163.70	91,774	143,986	0.02	161.99, ν = 0.265 Ref. [52] †

**Table 4:** The resonance frequencies ($f_{1}$, $f_{2}$) recovered from the vibration spectra obtained using the shear wave transducers and the corresponding Young’s moduli ($E_{1}$, $E_{2}$) retrieved using Equation (2). The final 3D FEM Young’s moduli (*E*) after adjustment to fit experimental resonance frequencies. The difference between $E_{2}$ and *E* is given in percentage. The † indicate ab initio calculated results found in the literature. The values of $K_{1}$ = 4.6356, $K_{2}$ = 7.3284, and the initial ν = 0.27.

Composition	Composition	$f_{1}$	$f_{2}$	Density	$E_{1}$	$E_{2}$	E (Gpa)	$F_{1}$ (Hz)	$F_{2}$ (Hz)	Difference Percentage	Reference
(wt. %)	(at. %)	(Hz)	(Hz)	(Kg/m^3^)	(GPa)	(Gpa)	3D FEM	3D FEM	3D FEM	$E_{2}$ − E (%)	E (GPa), ν
${\mathrm{Fe}}_{47}{\mathrm{Ti}}_{23}{\mathrm{Al}}_{30}$	${\mathrm{Fe}}_{35}{\mathrm{Ti}}_{20}{\mathrm{Al}}_{45}$	72,400	113,900	6469	184.80	183.10	184.80	72,512	137,700	0.92	
${\mathrm{Fe}}_{50}{\mathrm{Ti}}_{23}{\mathrm{Al}}_{27}$	${\mathrm{Fe}}_{38}{\mathrm{Ti}}_{20}{\mathrm{Al}}_{42}$	73,600	114,800	6580	192.20	189.10	192.20	73,000	114,770	1.61	
${\mathrm{Fe}}_{52}{\mathrm{Ti}}_{22}{\mathrm{Al}}_{26}$	${\mathrm{Fe}}_{40}{\mathrm{Ti}}_{20}{\mathrm{Al}}_{40}$	72,200	116,000	6682	189.90	196.10	189.90	73,900	115,936	3.26	
${\mathrm{Fe}}_{57}{\mathrm{Ti}}_{22}{\mathrm{Al}}_{21}$	${\mathrm{Fe}}_{45}{\mathrm{Ti}}_{20}{\mathrm{Al}}_{35}$	73,200	115700	6748	197.10	197.08	197.10	73,600	115,500	0.01	
${\mathrm{Fe}}_{63}{\mathrm{Ti}}_{29}{\mathrm{Al}}_{8}$	${\mathrm{Fe}}_{55}{\mathrm{Ti}}_{30}{\mathrm{Al}}_{15}$	72,700	114,800	6888	198.50	198.05	198.50	73,150	114,700	0.23	
${\mathrm{Fe}}_{65}{\mathrm{Ti}}_{20}{\mathrm{Al}}_{15}$	${\mathrm{Fe}}_{56}{\mathrm{Ti}}_{20}{\mathrm{Al}}_{24}$	72,600	115,000	6925	199.02	199.80	199.02	73,050	114,600	0.39	
${\mathrm{Fe}}_{68}{\mathrm{Ti}}_{24}{\mathrm{Al}}_{8}$	${\mathrm{Fe}}_{60}{\mathrm{Ti}}_{25}{\mathrm{Al}}_{15}$	72,800	116,800	6972	201.40	207.50	201.40	72,931	115,300	3.03	
${\mathrm{Fe}}_{78}{\mathrm{Ti}}_{14}{\mathrm{Al}}_{8}$	${\mathrm{Fe}}_{70}{\mathrm{Ti}}_{15}{\mathrm{Al}}_{15}$	72,600	114,700	7263	208.73	208.47	208.73	72,900	114,400	0.12	
${\mathrm{Fe}}_{80}{\mathrm{Al}}_{20}$	${\mathrm{Fe}}_{66}{\mathrm{Al}}_{34}$	70400	110,200	7412	200.03	196.30	200.03	70,650	110,800	1.86	200 Ref. [6]
${\mathrm{Fe}}_{60}{\mathrm{Al}}_{40}$	${\mathrm{Fe}}_{42}{\mathrm{Al}}_{58}$	82,400	130,200	5330	197.30	197.10	197.30	82,840	130,000	0.10	“
${\mathrm{Fe}}_{60}{\mathrm{Ti}}_{40}$	${\mathrm{Fe}}_{56}{\mathrm{Ti}}_{44}$	70,400	111400	7050	190.50	190.80	190.50	70740	111,000	0.16	191.66, ν = 0.287 Ref. [47] †
${\mathrm{Fe}}_{50}{\mathrm{Ti}}_{50}$	${\mathrm{Fe}}_{46}{\mathrm{Ti}}_{54}$	69,200	108,700	6950	181.47	179.16	181.47	69,174	108,700	1.27	182.38, ν = 0.28 Ref. [50] †
${\mathrm{Ti}}_{55}{\mathrm{Al}}_{45}$	${\mathrm{Ti}}_{40}{\mathrm{Al}}_{60}$	88,700	139,800	3880	166.45	165.40	166.45	88,521	139,270	0.63	160–176 Ref. [54]
${\mathrm{Ti}}_{45}{\mathrm{Al}}_{55}$	${\mathrm{Ti}}_{32}{\mathrm{Al}}_{68}$	90,800	145,700	3609	162.60	166.90	162.60	91,460	143,000	2.64	161.99, ν = 0.265 Ref. [52] †
